# Molecular mechanisms of cardioprotective effects mediated by transplanted cardiac ckit^+^ cells through the activation of an inflammatory hypoxia-dependent reparative response

**DOI:** 10.18632/oncotarget.22946

**Published:** 2017-12-06

**Authors:** Giovanni Puddighinu, Domenico D’Amario, Eleonora Foglio, Melissa Manchi, Andrea Siracusano, Elena Pontemezzo, Martina Cordella, Francesco Facchiano, Laura Pellegrini, Antonella Mangoni, Marco Tafani, Filippo Crea, Antonia Germani, Matteo Antonio Russo, Federica Limana

**Affiliations:** ^1^ Department of Experimental Medicine, Sapienza University of Rome, Rome, Italy; ^2^ Department of Cardiovascular Sciences, Catholic University of The Sacred Heart, Rome, Italy; ^3^ Department of Oncology and Molecular Medicine, Istituto Superiore di Sanità, Rome, Italy; ^4^ Department of Neurorehabilitation Sciences, Casa Cura Policlinico (CCP), Milan, Italy; ^5^ Department of Pathological Anatomy, Catholic University of The Sacred Heart, Rome, Italy; ^6^ Laboratory of Vascular Pathology, Istituto Dermopatico dell’Immacolata, IDI-IRCCS, Fondazione Luigi Maria Monti, Rome, Italy; ^7^ IRCCS San Raffaele Pisana, Rome, Italy; ^8^ MEBIC Consortium, San Raffaele Roma Open University, Rome, Italy; ^9^ San Raffaele Roma Open University, Rome, Italy

**Keywords:** ckit^+^ stem cells and hypoxia, inflammatory and reparative response, myocardial infarction, molecular rehabilitation, cardiac repair

## Abstract

The regenerative effects of cardiac ckit^+^ stem cells (ckit^+^CSCs) in acute myocardial infarction (MI) have been studied extensively, but how these cells exert a protective effect on cardiomyocytes is not well known. Growing evidences suggest that in adult stem cells injury triggers inflammatory signaling pathways which control tissue repair and regeneration. Aim of the present study was to determine the mechanisms underlying the cardioprotective effects of ckit^+^CSCs following transplantation in a murine model of MI.

Following isolation and *in vitro* expansion, cardiac ckit^+^CSCs were subjected to normoxic and hypoxic conditions and assessed at different time points. These cells adapted to hypoxia as showed by the activation of HIF-1α and the expression of a number of genes, such as VEGF, GLUT1, EPO, HKII and, importantly, of alarmin receptors, such as RAGE, P2X7R, TLR2 and TLR4. Activation of these receptors determined an NFkB-dependent inflammatory and reparative gene response (IRR). Importantly, hypoxic ckit^+^CSCs increased the secretion of the survival growth factors IGF-1 and HGF. To verify whether activation of the IRR in a hypoxic microenvironment could exert a beneficial effect *in vivo*, autologous ckit^+^CSCs were transplanted into mouse heart following MI. Interestingly, transplantation of ckit^+^CSCs lowered apoptotic rates and induced autophagy in the peri-infarct area; further, it reduced hypertrophy and fibrosis and, most importantly, improved cardiac function.

ckit^+^CSCs are able to adapt to a hypoxic environment and activate an inflammatory and reparative response that could account, at least in part, for a protective effect on stressed cardiomyocytes following transplantation in the infarcted heart.

## INTRODUCTION

Myocardial infarction (MI) represents a leading cause of morbidity and mortality worldwide [1].

Although the ongoing therapeutic strategies, represented by prompt reperfusion of the ischemic myocardium, and pharmacological treatments, mainly based on anti-platelet agents, statins, ACE inhibitors and beta blockers [2], have contributed to decrease the mortality of patients, cardiac ischemia-reperfusion (I/R) injury and adverse remodeling still remain the major factors impairing cardiac function and determining an unsatisfactory prognosis [3]. A better understanding of the pathophysiology of these conditions could help in the identification of new therapeutic strategies.

MI is associated with a potent inflammatory response (reviewed in [4]). During cardiac ischemia, the shortage of oxygen and nutrients leads to the death of cardiomyocytes and endothelial cells [5] with the following passive release of endogenous damage-associated molecular pattern molecules (DAMPs), also known as alarmins, that activate the local macrophages and promote the recruitment of leukocytes [6]. In the surviving cardiomyocytes and endothelial cells, as well as in the inflammatory cells, the post-MI hypoxic microenvironment induces the activation of the hypoxia inducible factor 1 alpha (HIF-1 α) [7], allowing the adaptation of these cells to the reduced oxygen level, and promoting the acquisition of an inflammatory reparative phenotype through the expression of a wide range of genes [8-11], among which the receptors for alarmins [12-17]. The binding of the alarmins their receptors determine the activation of the nuclear factor kappa-light-chain-enhancer of activated B cells (NFkB) and the expression of hundreds of genes involved in the inflammatory reparative response (IRR) [18] and accountable for the inflammatory cascade observed following the MI [19]. Of note, HIF-dependent adaptation and expression of proinflammatory genes has been also observed in hypoxic stem cells [20].

The observation that, in several other cardiac pathologies, the immune response was accountable for extended cardiac injury and accentuated adverse remodeling has led to the investigation of different strategies aimed to suppress the inflammatory response in post-MI. However due to the detrimental effects of these approaches on the cardiac function [21-24], treatments with broad immunosuppressive drugs have been largely abandoned. Results of these experiments indicated that the inflammatory response is necessary to eliminate debris and to initiate the reparative process, but it needs to be tightly regulated to prevent a pathological remodeling of the myocardium which may led to congestive heart failure. In particular, prolonged or excessive induction of proinflammatory signalling has been linked to apoptosis of cardiomyocytes, enhanced matrix-degrading processes and extended fibrotic changes beyond the initial infarct [25]. Hence, an effective therapy should not interfere with the activation of the inflammatory pathway but rather reduce the length and damage of the response.

In this study, we aimed to test the hypothesis that cardiac c-kit^+^ stem cells (ckit^+^CSCs), whose ability to modulate the inflammatory response was recently demonstrated [26], acquire an inflammatory phenotype under hypoxic conditions that could allow them to modulate the reparative response and reduce the damage in a hypoxic tissue, as the infarcted heart, following transplantation. In particular, *in vitro*, following hypoxia treatment, we observed a significant up-regulation of the IRR genes in ckit^+^CSCs. Further, after their injection in a mouse model of MI, we observed a reduced level of apoptosis together with an induction of autophagy and an attenuation in cardiac remodeling. These effects were associated to an improvement in cardiac performance.

## RESULTS

### Hypoxia regulates expression of pro-inflammatory genes and proteins in ckit^+^CSCs

Several evidences suggest that in a hypoxic tissue, inflammation can be activated causing damage as well as repair. This response, named IRR, can occur in many important human pathologies and involve chiefly distant and resident leukocytes. Nevertheless, HIF-dependent adaptation and expression of pro-inflammatory genes has been also observed in hypoxic stem cells and progenitors [20].

To verify whether ckit^+^CSCs are able to adapt to a hypoxic environment by the IRR, these cells were cultured and incubated either in normoxia or hypoxia for 6, 24, and 48 hours (h).

**Figure 1:**
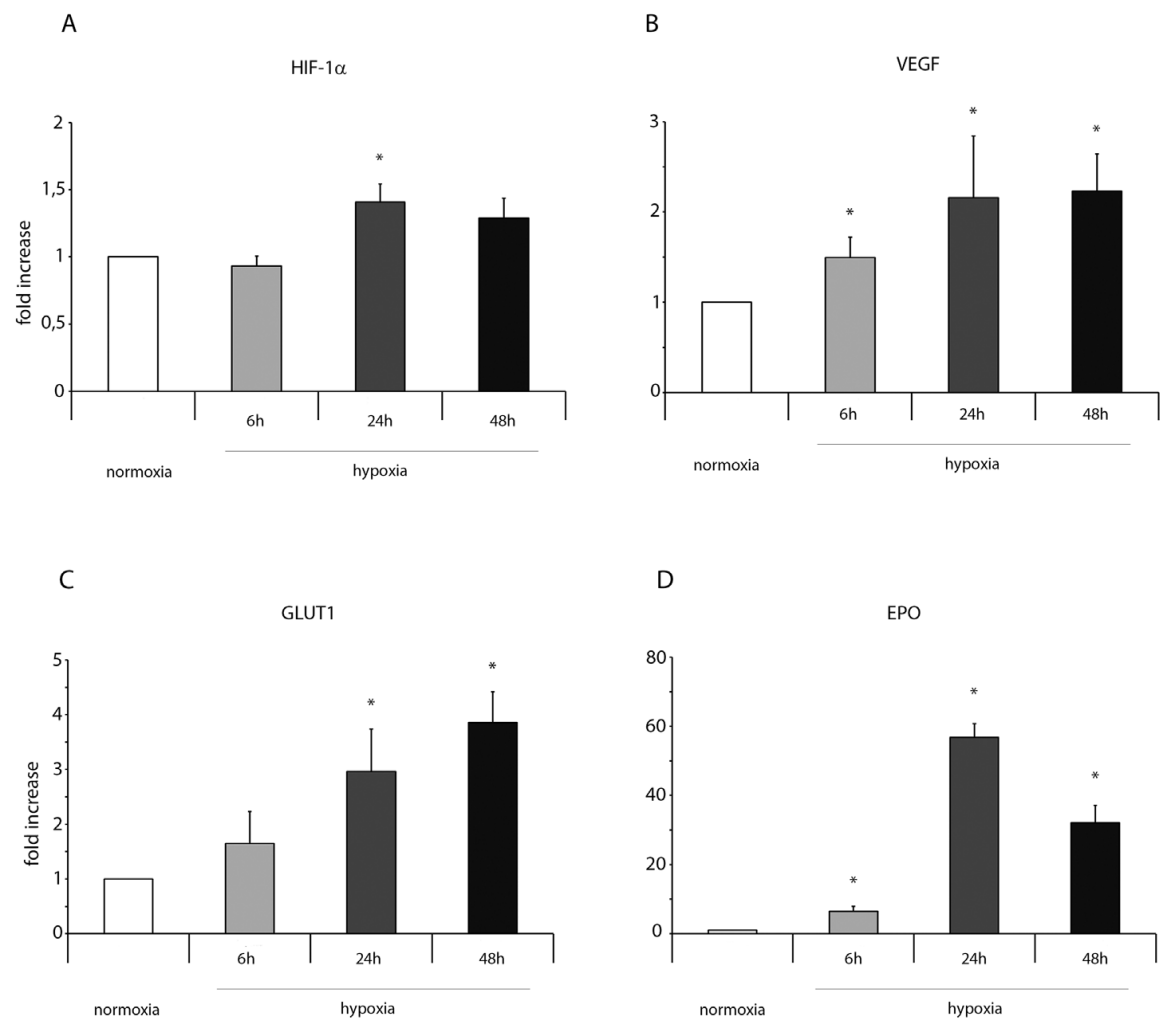
Hypoxia enhances mRNA expression of HIF-1 α and its target genes in ckit^+^CSCs Ckit^+^CSCs were incubated under normoxic or hypoxic conditions for 6, 24 and 48h. mRNA expression levels of **(A)** HIF-1 α, **(B)** VEGF, **(C)** GLUT1 and **(D)** EPO were determined by real time PCR. The bar graphs show fold increases in expression of the studied genes in ckit^+^CSCs with respect to control normoxic cells, set at 1. Data were normalized to GUSB, a housekeeping gene, and represent means ± SEM for three separate experiments, each repeated in triplicate. ^*^, P < 0.05.

**Figure 2:**
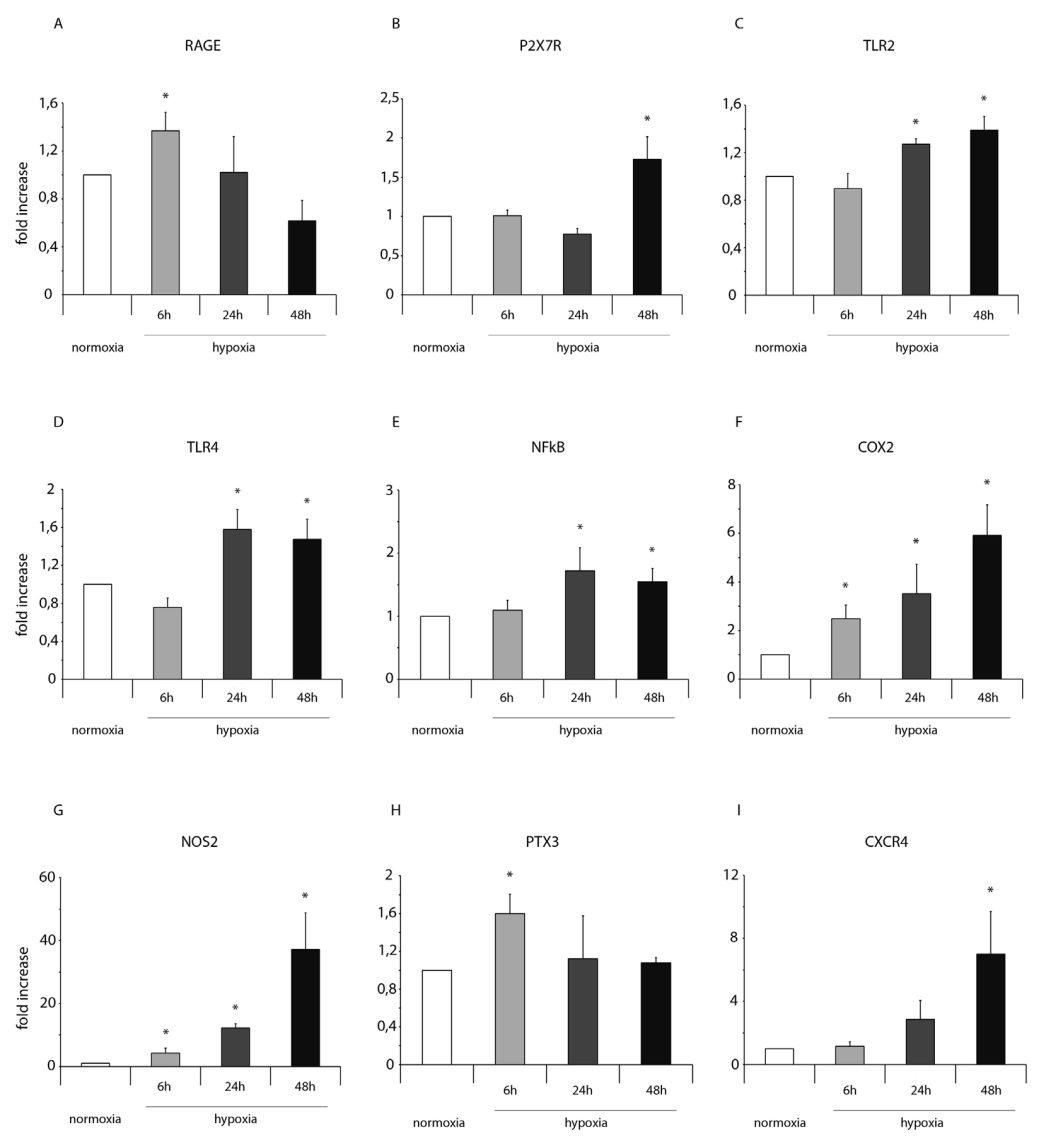
Hypoxia enhances mRNA expression of NFkB and markers of the NFkB-dependent inflammatory and reparative response in ckit^+^CSCs Ckit^+^CSCs were incubated under normoxic and hypoxic conditions for 6, 24 and 48h. mRNA expression levels for **(A)** RAGE, **(B)** P2X7R, **(C)** TLR2, **(D)** TLR4, **(E)** NFkB, **(F)** COX-2, **(G)** NOS2, **(H)** PTX3 and **(I)** CXCR4 were determined by real time PCR. The bar graphs show fold increases in expression of the studied genes in ckit^+^CSCs with respect to control normoxic cells, set at 1. Data were normalized to GUSB, a housekeeping gene, and represent means ± SEM for three separate experiments, each repeated in triplicate. ^*^, P < 0.05.

Importantly, in normoxic condition, ckit^+^CSCs examined in our study did not show any significant presence of leukocytes as shown by CD45 FACS analysis (Supplementary Figure 1). Hypoxia clearly influenced the expression of pro-inflammatory genes and proteins. HIF-1 α mRNA expression was significantly increased following 24h of hypoxia (Figure 1A) while its target gene VEGF significantly increased at all three time points but mainly at 24 and 48h (Figure 1B). Among the other HIF-1 α target genes, as showed by RT-PCR, the expression level of GLUT1 gradually increased from 6 to 48h (Figure 1C) while erythropoietin (EPO) expression strongly increased at all three time points showing a peak after 24h of hypoxia (Figure 1D). Importantly, alarmin receptors such as RAGE, P2X7R, the Toll-like receptors 2 and 4 (TLR2 and TLR4) were activated by hypoxia. The membrane receptor RAGE showed a transient induction at 6h and a low induction by hypoxia at 24 and 48h (Figure 2A). The expression of the other membrane receptor P2X7R was increased only following 48h of hypoxia (Figure 2B). TLR2 and TLR4 showed a similar expression pattern: a decrease at 6h and a significant increase both at 24 and 48h (Figure 2C-2D).

The activation of these receptors resulted in increased expression levels of NFkB (Figure 2E) that led to the expression of genes belonging to the family of the IRR (all involved in defense, protection and repair of cells and tissues of the IRR as well) as the pro-inflammatory proteins cyclooxygenase-2 (COX2), the pentraxin-3 (PTX3), the chemokine (C-X-C motif) receptor 4 (CXCR4) and the nitric oxide synthase-2 (NOS2). In particular, hypoxia increased the expression of the inducible enzymes COX2 (Figure 2F) and NOS2 (Figure 2G) mainly at 48h but significantly at all three time points. Conversely, the expression of PTX3 was induced by hypoxic conditions only at 6h (Figure 2H). Finally, CXCR4 was induced by hypoxia at 48h (Figure 2I). However, in all experimental conditions, we always observed an increased expression of the genes related to HIF-1 α and NFkB in CSCs under hypoxia compared to normoxia.

**Figure 3:**
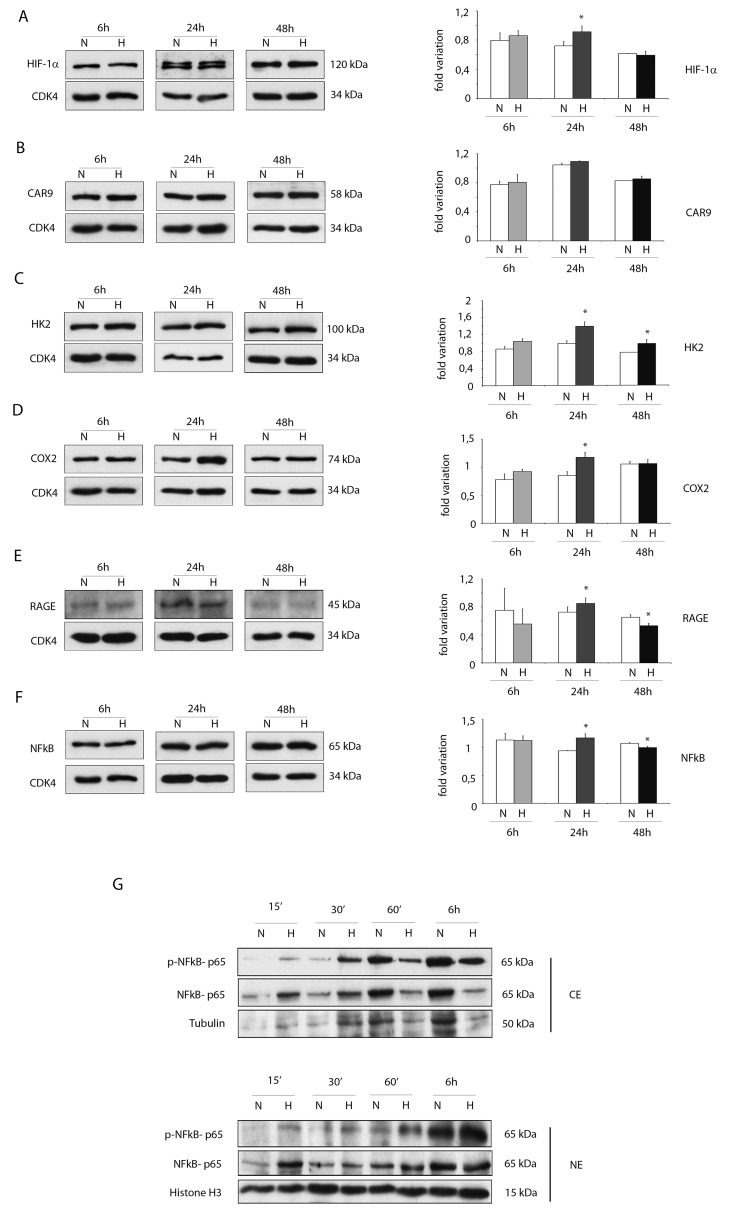
Hypoxia enhances protein expressions of pro-inflammatory proteins in ckit^+^CSCs Western blot analysis showing the expression of **(A)** HIF-1 α, **(B)** CAR9 and **(C)** HK2, **(D)** COX2 and **(E)** RAGE and **(F)** NFkB in ckit^+^CSCs under hypoxic conditions (H) at 6, 24 and 48h compared to normoxic conditions (N). The same filter was probed with anti-CDK4 pAb to show the equal loading. Left panel: A representative Western blotting of three independent experiments is shown. Right panel: Densitometric analysis of Western blot. Data are shown as means ± SEM. ^*^P < 0.05 vs normoxic conditions (N). **(G)** Western blot analysis showing the expression of phospho-specific anti-NFkB p65, anti-NFkB p65, tubulin and histone H3. The analysis was performed using cytoplasmic (CE) and nuclear (NE) extracts prepared from ckit^+^CSCs under hypoxic conditions (H) at 15’, 30’, 60’ and 6h compared to normoxic conditions (N). A representative Western blotting of three independent experiments is shown.

For several markers, increased protein expression levels were obtained by WB analysis under hypoxic conditions mainly at 24h (Figure 3A-3F).

**Figure 4:**
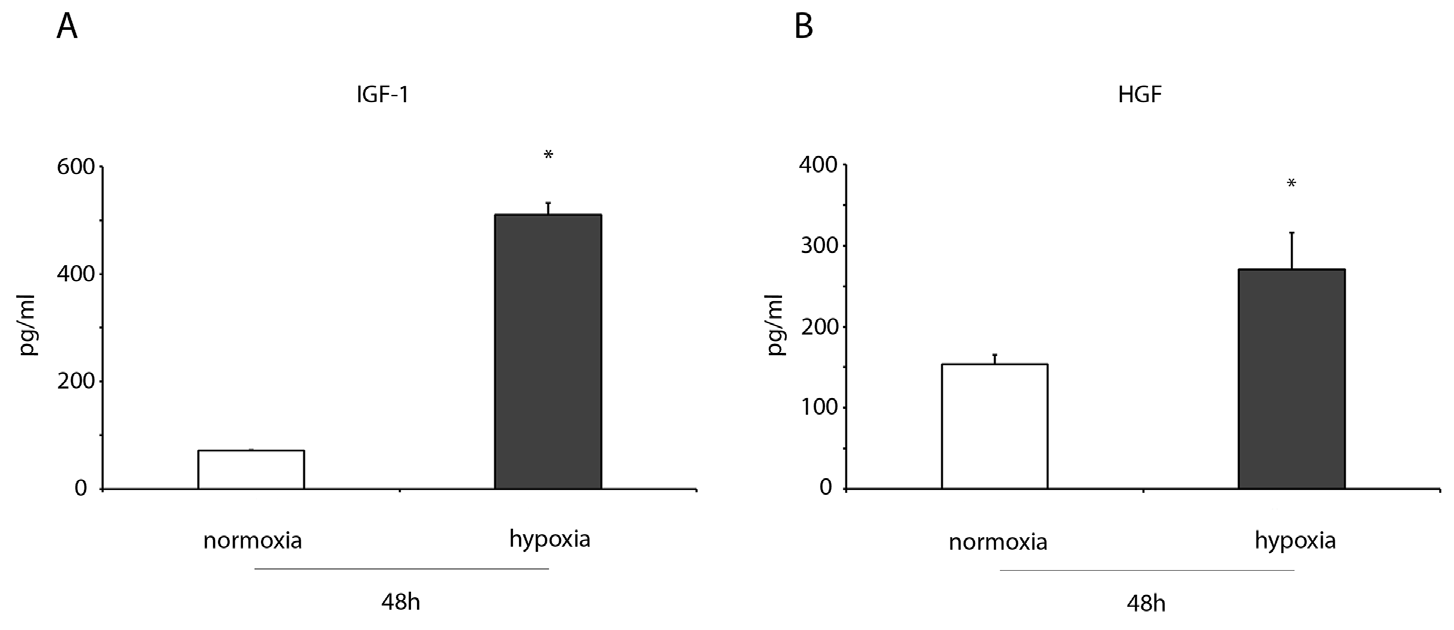
IGF-1 and HGF levels increase in the conditioned medium of hypoxic ckit^+^CSCs ELISA detection of **(A)** IGF-1, **(B)** HGF in in the conditioned medium of 48h normoxic and hypoxic ckit^+^CSCs (n= 3 independent experiments). Results are presented as means ± SD. ^*^P < 0.001 vs normoxic conditions.

In particular, hypoxia increased the expression of HIF-1 α at 24h (Figure 3A). Noteworthy, the protein expression of the transporter carbonic anhydrase 9 (CAR9) was slightly increased at 6 and 24h while the metabolic enzyme hexokinase 2 (HK2), regulated by HIF-1 α as CAR9, was significantly increased following 24h and 48h of hypoxia (Figure 3B and 3C). The expression of COX2 and RAGE was increased after 24h of hypoxia but the expression of RAGE was significantly reduced after 48h of hypoxia (Figure 3D and 3E). Hypoxia increased the expression of NFkB p65 subunit (NFkB p65) at 24h (Figure 3F) and, most importantly, induced its activation as showed in Figure 4G. Specifically, Western blot analysis showed that hypoxia induced the phosphorylation of cytoplasmic NFkB p65 within the first 30 min and its nuclear translocation in a time-dependent manner (Figure 3G).

### ckit^+^CSCs enhance the production of IGF-1 and HGF under hypoxia conditions

ELISA assays were performed to measure the levels of two growth factors, both involved in cardioprotection, in the conditioned medium of normoxic and hypoxic ckit^+^CSCs. After 48h of hypoxia, we detected a significant increase in IGF-1 (Figure 4A) and HGF levels (Figure 4B) in conditioned media of hypoxic ckit^+^CSCs compared to normoxia.

### ckit^+^CSCs enhance IL10 circulating levels and protein expression

**Figure 5:**
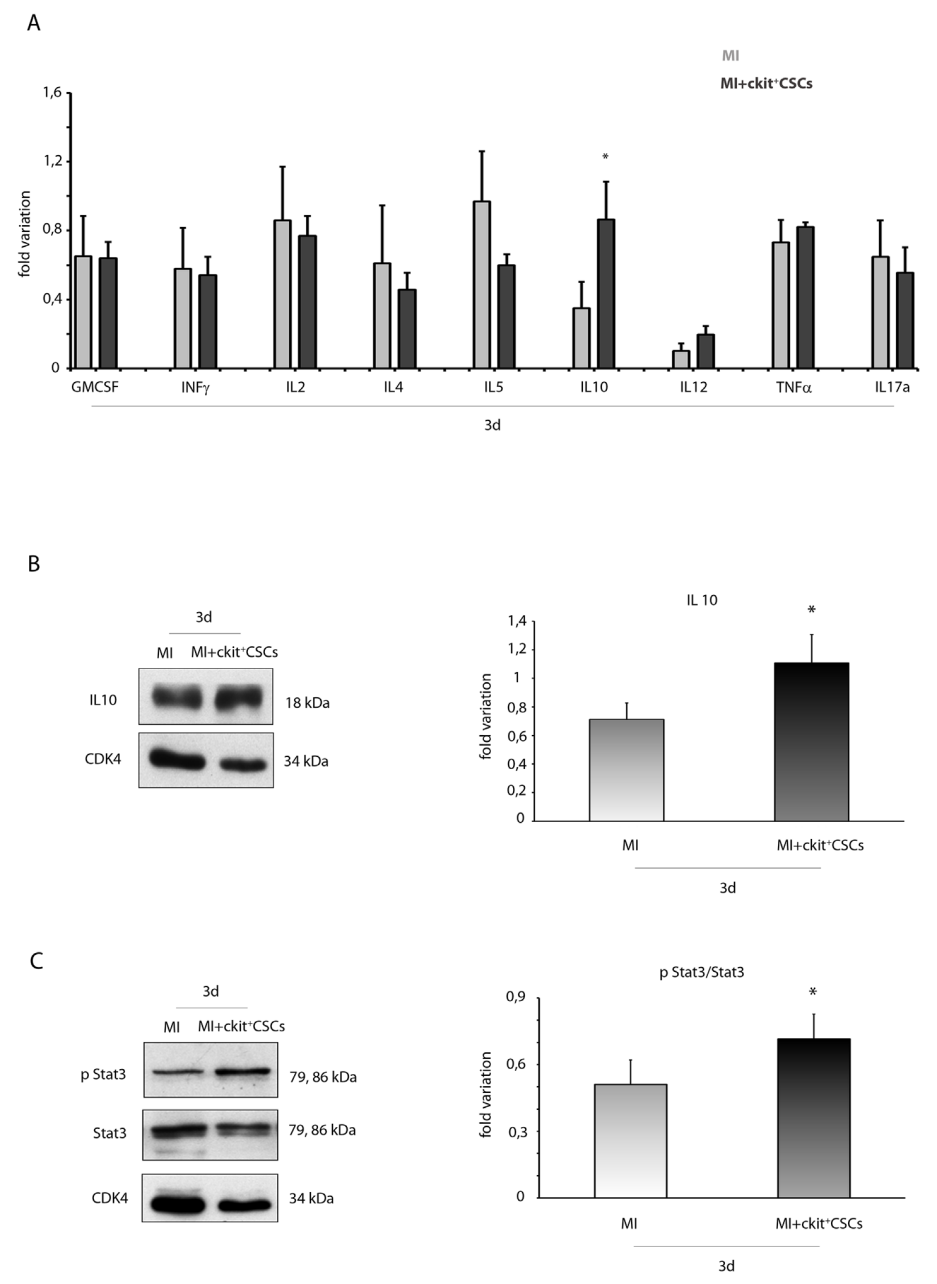
ckit^+^CSCs enhance circulating levels and protein expression of IL10 in infarcted hearts following transplantation **(A)** Circulating levels of pro- and anti-inflammatory cytokines detected by Luminex assay 3 days following surgery in the serum of PBS treated infarcted mice used as controls (MI, n=4) and ckit^+^CSC transplanted infarcted mice (MI+ckit^+^CSCs, n=4), all normalized for their relative sham controls. Data are shown as means ± SEM. ^*^P<0.05 vs MI. Western blot analysis showing the expression of **(B)** IL10 and **(C)** phospho-STA3/STAT3 in the infarcted region of 3 day-infarcted heart following ckit^+^CSC transplantation (MI+ckit^+^CSCs, n=3). Infarcted hearts treated with PBS were used as control (MI, n=3). Left panel: A representative Western blotting of three independent experiments is shown. Right panel: Densitometric analysis of Western blot. Data are shown as means ± SEM. ^*^P < 0.05 vs untreated myocardial infarction (MI).

Since activation of the IRR leads to protection and repair of damaged cells, we hypothesized that following transplantation into the infarcted myocardium, ckit^+^CSCs would exert a cardioprotective effect by activating this pathway. To test this hypothesis, ckit^+^CSCs were transplanted into the damaged heart of an ischemic mouse. Firstly, by Luminex assay, we detected circulating levels of different pro- and anti-inflammatory cytokines 3 days following surgery: we did not find significant differences among injected infarcted hearts and control infarcted hearts except for IL10, a potent anti-inflammatory cytokine, that resulted significantly up regulated in the ckit^+^CSC treated group (Figure 5A). This result was confirmed by WB analysis on the infarcted region of 3 day-infarcted hearts: we detected significantly enhanced expression levels of IL10 in ckit^+^CSC transplanted hearts compared to control hearts (Figure 5B). Further, comparing the samples from the same groups of animals, we also detected an increased phosphorylation of STAT3 following infarction and transplantation (Figure 5C).

### ckit^+^CSCs improve cardiac function 2 weeks following MI

**Figure 6:**
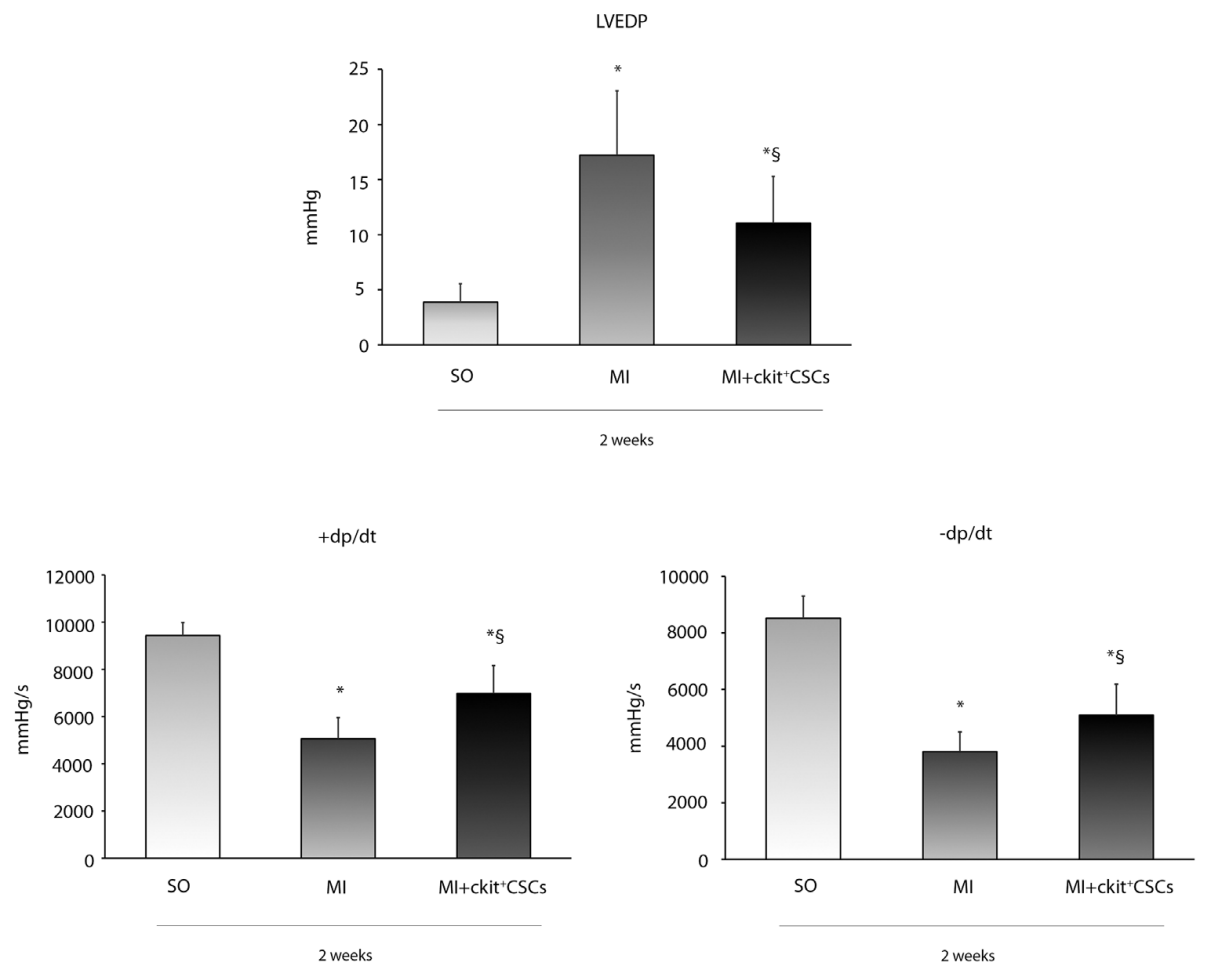
Effects of ckit^+^CSCs on cardiac function Functional assessment of myocardial function, hemodynamic parameters. LVEDP, LV +dP/dt (rate of pressure rise) and LV -dP/dt (rate of pressure decay) in sham-operated (SO) and infarcted mice treated either with PBS (MI) or ckit^+^CSCs (MI+ckit^+^CSCs). Measurements were obtained 14 days after myocardial infarction and ckit^+^CSC transplantation; Data represent means±SD (SO, n=8; MI, n=7; MI+ckit^+^CSCs, n=7; ^*^p<0.05 *vs* SO; ^§^p<0.05 *vs* MI).

We investigated whether ckit^+^CSC transplantation was associated to an improvement in cardiac function following MI. These cells were injected in the border zone of mice immediately after the induction of MI. Mice receiving intramyocardial injection of PBS just after MI were used as controls. Hemodynamic measurements were performed 14 days after infarction. Ckit^+^CSC transplanted mice showed better preservation of myocardial function in comparison to control mice: left ventricular end diastolic pressure (LVEDP) was lower (11±4.2 mmHg *vs* 17±5.8 mmHg) (Figure 6), while both positive dP/dt (rate of pressure rise) and negative dP/dt (rate of pressure decay) were higher suggesting a less severe damage in the presence of treatment.

### ckit^+^CSCs improve left ventricular remodeling

**Figure 7:**
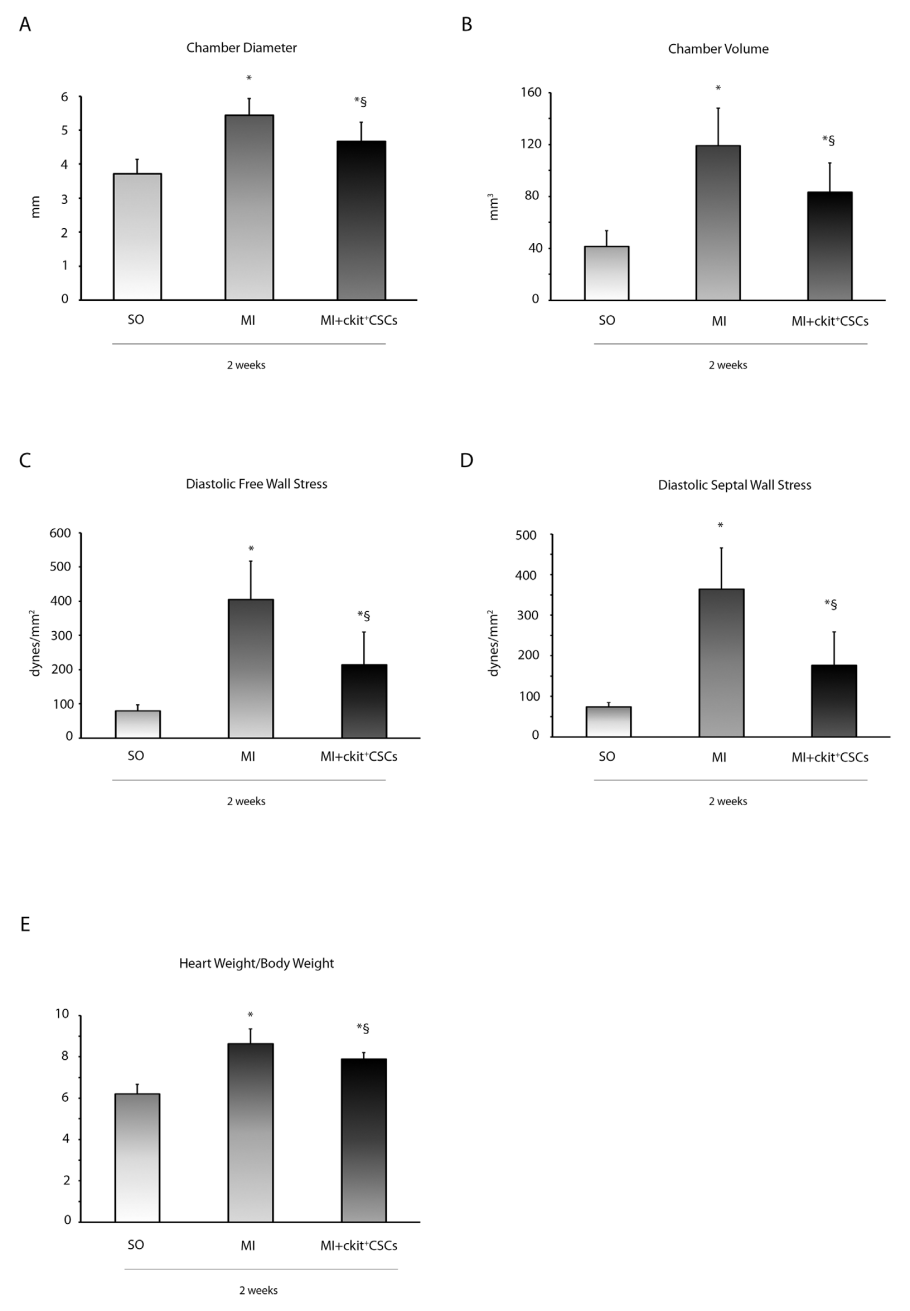
ckit^+^CSCs improve left ventricular remodeling **(A)** LV chamber diameter, **(B)** LV chamber volume, **(C)** diastolic free wall stress, **(D)** diastolic septal wall stress and **(E)** Heart weight/Body weight following MI. All measurements were obtained 2 weeks after ckit^+^CSC or PBS treatment. Results are presented as mean±SD (SO, n=7; MI, n=6; MI+ckit^+^CSCs, n=7; ^*^p<0.05 *vs* SO; ^§^p<0.05 *vs* MI).

The functional improvement in ckit^+^CSC transplanted hearts was associated with reduced LV chamber diameter and volume (Figure 7A and 7B). The attenuation in ventricular dilation together with the hemodynamic parameters resulted in a reduction in diastolic free wall and septal wall stress (Figure 7C and 7D). Further, Heart weight/Body weight ratio, an index of cardiac hypertrophy, was reduced in transplanted hearts compared to controls (Figure 7E).

### ckit^+^CSCs attenuate hypertrophy in the infarcted murine heart

**Figure 8:**
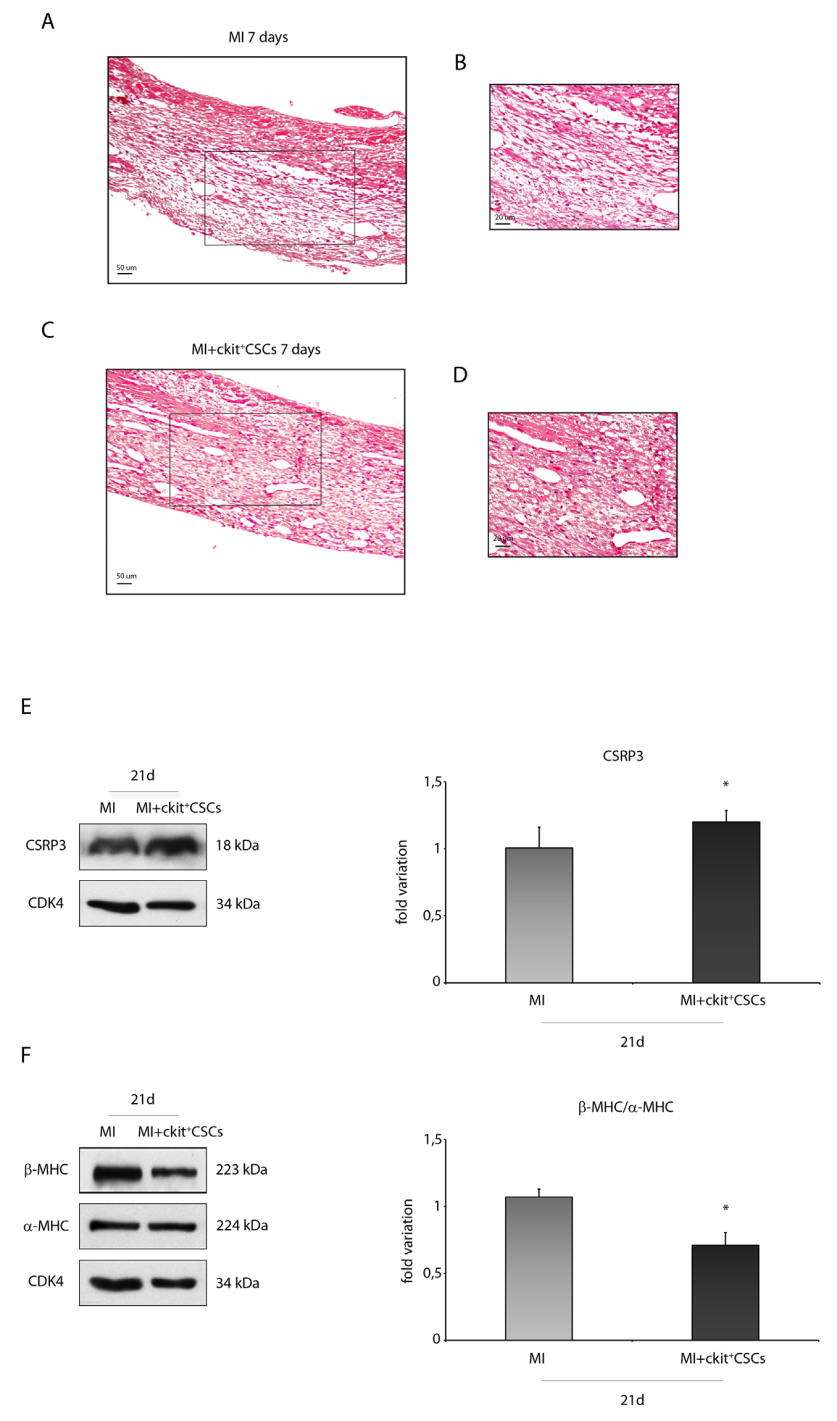
ckit^+^CSCs attenuate hypertrophy following acute MI Representative H&E stained myocardial sections **(A-D)** of 7 day infarcted region from control (MI) (A) and ckit^+^CSC transplanted hearts (C). (B) and (D) represent high magnification of the insets shown in (A) and (C), respectively. The relative levels of **(E)** CSRP3 and **(F)** β-MHC/ α-MHC were investigated in the border zone of 21 day-infarcted control hearts (MI, n=3) or ckit^+^CSC transplanted hearts (MI+ckit^+^CSCs, n=3) by Western blot (left panel) and relative densitometry of three independent experiments (right panel). Data are shown as means ± SEM. ^*^P < 0.05 vs MI.

Hematoxylin-eosin (H&E) staining showed that the scar from ckit^+^CSC transplanted hearts displayed a better preservation compared with that from control hearts suggesting a beneficial effect of cell therapy 7 days following MI (Figure 8A-8D). Further, markers of hypertrophy in the survived myocardium of both groups of mice were analyzed at a later time point. In particular, muscle LIM Protein (MLP), also known as cysteine rich protein 3 (CSRP3, CRP3), has a significant impact on cardiac muscle physiology and pathology. Specifically, in mice, reduced expression of CSRP3 leads to myocardial hypertrophy followed by dilated cardiomyopathy and heart failure [27]. In our experimental model, by WB analysis, we detected a higher expression level of CSRP3 in the border zone of 21 day-infarcted hearts following ckit^+^CSC transplantation compared to controls (Figure 8E).

Cardiac hypertrophy is characterized by a switch of expression from α-myosin heavy chain (α-MHC) to β-MHC [28, 29]. Following 21 days of cell therapy, we observed, in the border zone of infarcted hearts, the presence of lower levels of β-MHC and of higher levels of α-MHC, as showed by WB, leading to a marked decrease in MHC β-α ratio (Figure 8F).

### ckit^+^CSCs reduce cardiac fibrosis following acute MI

**Figure 9:**
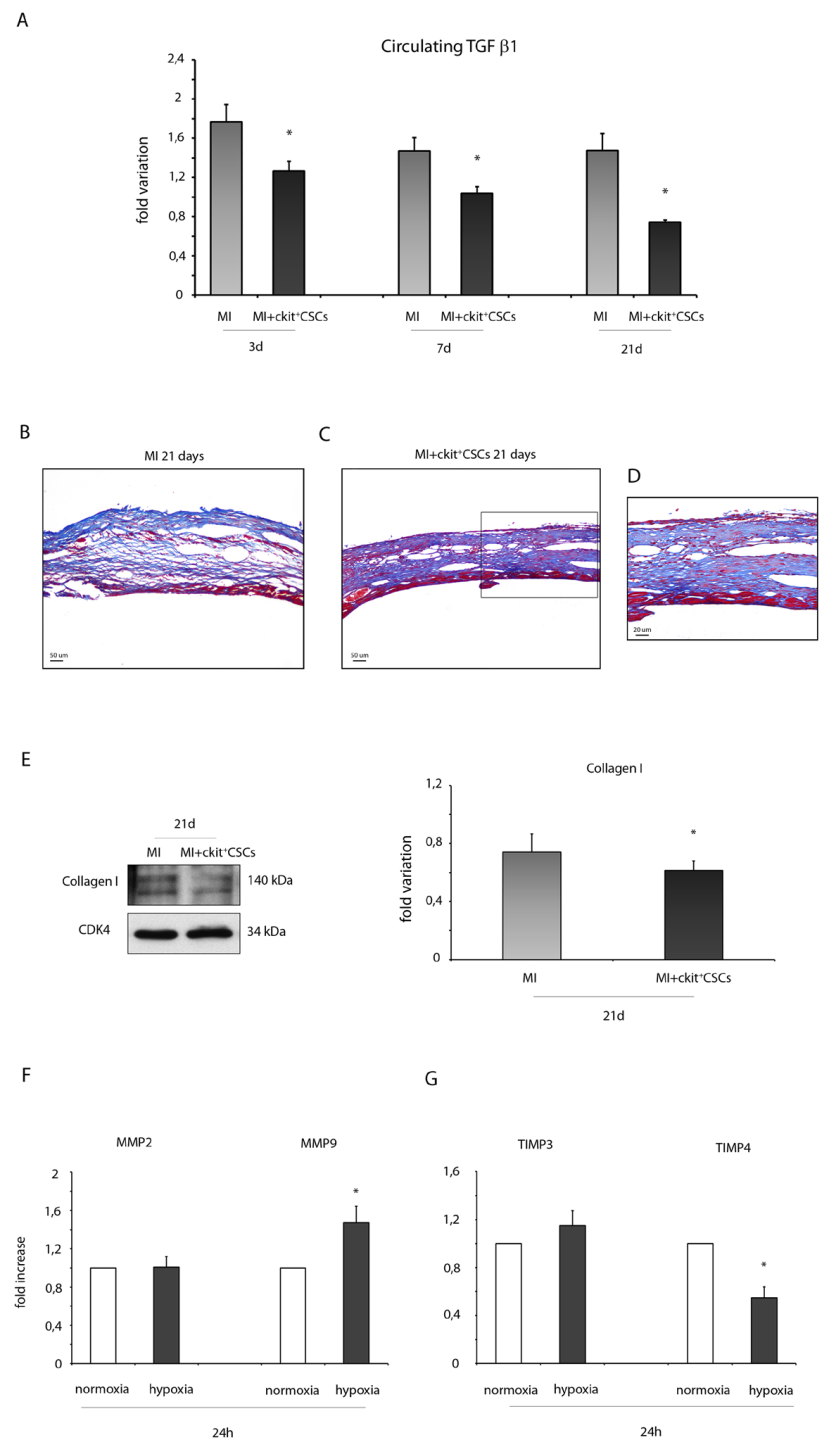
ckit^+^CSCs reduce the expression of fibrotic markers **(A)** Circulating levels of TGF β1 detected by Luminex assay 3, 7 and 21 days following surgery in the serum of PBS treated infarcted mice used as controls (MI, n=4) and ckit^+^CSC transplanted infarcted mice (MI+ckit^+^CSCs, n=4), all normalized for their controls. Data are shown as means ± SEM. ^*^P<0.05 vs MI. **(B-D)** Representative Azan-Mallory staining photomicrographs of control (MI) (B) and ckit^+^CSC transplanted infarcted hearts (MI+ckit^+^CSCs) (C, D) depicting fibrosis (blue area), 21 days after treatment. (D) represents high magnification of the insets shown in (C). **(E)** Collagen I expression in 21 day-infarcted control hearts (MI, n=3) or ckit^+^CSC transplanted hearts (MI+ckit^+^CSCs, n=3) by Western blot (left panel) and relative densitometry of 3 independent experiments (right panel). Data are shown as means ± SEM. ^*^P < 0.05 vs MI. **(F)** MMP2 and MMP9, **(G)** TIMP3 and TIMP4 mRNA expression by real time PCR in normoxic and hypoxic ckit^+^CSCs at 24h. Data were normalized to GUSB, a housekeeping gene and represent means ±SEM; Data were obtained from 3 independent experiments. ^*^p< 0.02 vs normoxic ckit^+^CSCs.

TGF β is an indicator of cardiac fibrosis that plays an important role in the pathogenesis of cardiac remodeling. In our experimental model, 3, 7 and 21 days following ckit^+^CSC treatment, infarcted mice were characterized by circulating levels of TGF β1, detected by Luminex assay, significantly lower than that in controls suggesting an attenuation in adverse remodeling (Figure 9A).

In the infarcted murine heart, TGF β promotes myofibroblast transdifferentiation and matrix synthesis. Accordingly to our results on circulating TGF β1, we detected lower collagen deposition as showed by Azan –Mallory staining (Figure 9B-9D) and by a reduced protein expression of collagen I in the infarcted hearts 21 days following stem cell transplantation compared to control hearts (Figure 9E). Further, in order to account for these results, we evaluated whether hypoxic ckit^+^CSCs had any effect on the expression of matrix metalloproteinases (MMPs) and their inhibitors (TIMPs). 24h of hypoxia had no significant effects on MMP2 mRNA while it markedly enhanced MMP9 mRNA in ckit^+^CSCs (Figure 9F). Additionally, when we examined the mRNA expression of the MMP inhibitors, we found that TIMP4 was significantly lower in hypoxic ckit^+^CSCs than in normoxic cells (Figure 9G). In contrast, there was no effect on TIMP3 expression (Figure 9G).

### ckit^+^CSCs reduce apoptosis and induce autophagy *in vivo* following acute MI

**Figure 10:**
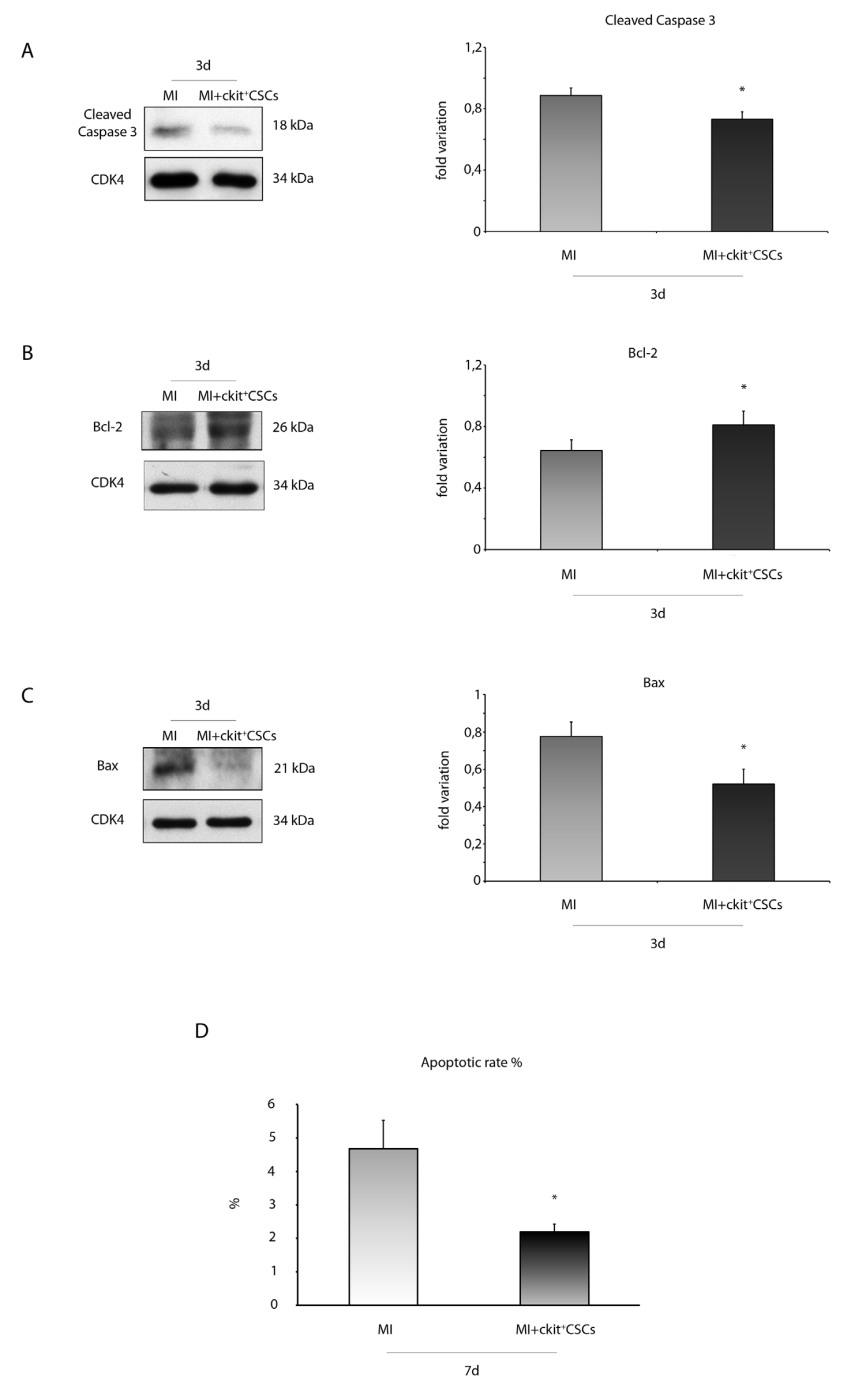
ckit^+^CSCs modulate the expression of apoptotic markers in treated infarcted hearts Western blot analysis showing the expression of **(A)** cleaved-caspase 3, **(B)** Bcl-2 and **(C)** Bax, in the border zone of 3 day-infarcted control hearts (MI, n=3) or ckit^+^CSC transplanted hearts (MI+ckit^+^CSCs, n=3). Left panel: A representative Western blotting of three independent experiments is shown. Right panel: Densitometric analysis of Western blot. Data are shown as means ± SEM. ^*^P < 0.05 vs MI. **(D)** Cardiomyocyte apoptosis measured by TdT assay. Bar graph showing apoptotic rate in the peri-infarct area of the myocardium of control (MI) and ckit^+^CSC transplanted infarcted (MI+ckit^+^CSCs) hearts. Data represent means±SD (MI, n=5; MI+ckit^+^CSCs, n=5; ^*^p<0.0052 *vs* MI).

We verified the anti-apoptotic effect of transplanted ckit^+^CSCs 3 days following MI. Specifically, we evaluated caspase-3 cleavage by WB analysis in the border zone of infarcted hearts (Figure 10A). Treated hearts showed lower expression levels of cleaved caspase-3 compared to controls. Further, we also measured the expression levels of apoptotic downstream targets, including the anti-apoptotic protein Bcl-2 as well as the pro-apoptotic protein Bax (Figure 10B-10C). Results showed that the protein expression of Bcl-2 was significantly increased in the border zone of 3 day-infarcted hearts by transplantation (Figure 10B). Conversely, cell treatment significantly inhibited Bax expression compared to the control group (Figure 10C).

Importantly, these results were confirmed by the measurement of apoptotic rate 7 days following MI and transplantation. Apoptosis in the peri-infarct region was significantly lower in transplanted animals compared to controls with nearly a 2 fold reduction in apoptotic rate (2.27 ± 0.22% *vs* 4.7 ± 0.84%, p= 0.0052) as showed by quantification of apoptotic cardiomyocytes (Figure 10D).

**Figure 11:**
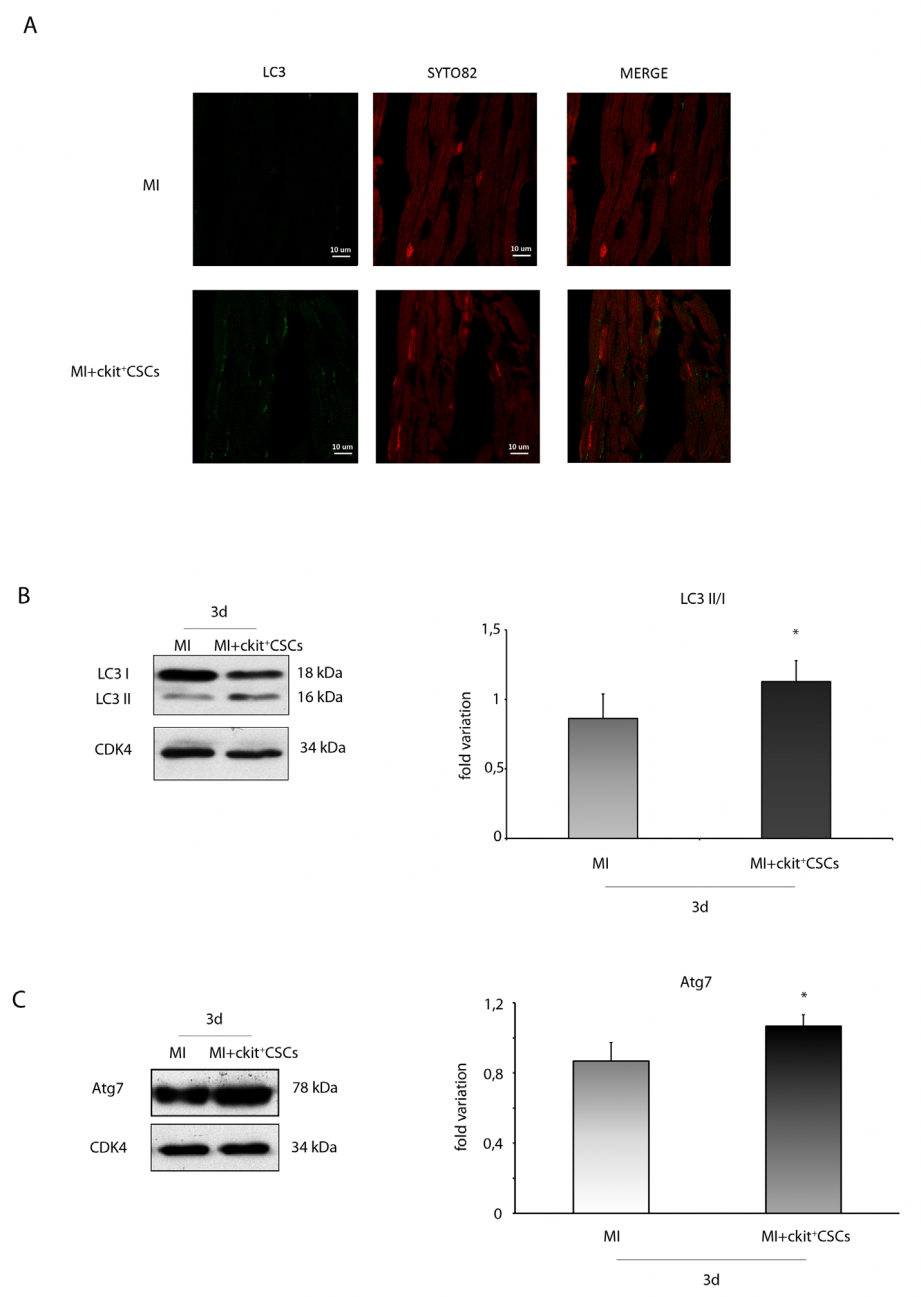
ckit^+^CSCs modulate the expression of autophagic markers in treated infarcted hearts Representative images of immunofluorescence for LC3 **(A)** of heart tissue sections from 3 day-infarcted control hearts (MI) or ckit^+^CSC transplanted hearts (MI+ckit^+^CSCs). Left panel: green fluorescence indicates LC3 labeling. Central panel: orange fluorescence indicates SYTO 82 Orange Fluorescent staining of nuclei. Right panel: merge of both images. Western blot analysis showing the expression of **(B)** LC3 and **(C)** Atg7, in the border zone of 3 day-infarcted control hearts (MI, n=3) or ckit^+^CSC transplanted hearts (MI+ckit^+^CSCs, n=3). Left panel: A representative Western blotting of three independent experiments is shown. Right panel: Densitometric analysis of Western blot. Data are shown as means ± SEM. ^*^P < 0.05 vs MI.

Since recent studies have indicated a protective role of the autophagic process in ischemic heart disease [30, 31], we tested whether reduced apoptotic rate in the peri-infarct region of transplanted hearts was associated to increased autophagy. We firstly performed an immunofluorescence for the ubiquitin-like modifier LC3 (LC3). Results revealed that, 3 days following MI, the ckit^+^CSC transplanted group exhibited an higher number of cardiomyocytes with LC3 punctate dots compared to the control group. To further confirm that autophagy was induced, we analyzed the expression of LC3 by WB analysis (Figure 11A). Results showed that, 3 days following MI, there was a significant increase in LC3 II formation in the border zone of ckit^+^CSC transplanted hearts compared to controls indicative of autophagosome formation. Further, the infarcted hearts were also characterized, following cell transplantation, by significant up-regulation of autophagy-related gene 7 (Atg7) protein level, another important protein for autophagosome formation (Figure 11B).

## DISCUSSION

In the present study, we have demonstrated that cardiac ckit^+^CSCs adapt to hypoxia and acquire an inflammatory reparative phenotype through the expression of HIF-1α and NFkB target genes and the release of growth factors. In *vivo*, following intramyocardial injection of these cells, ischemia adaptation led to cardioprotection of the infarcted myocardium. Specifically, ckit^+^CSC transplantation after acute MI lowered apoptosis, induced autophagy and attenuated adverse remodeling by reducing hyperthophy and fibrosis. These effects contributed to an improvement in cardiac performance.

The regenerative capacity of cardiac ckit^+^CSCs in acute MI has been studied extensively but is highly debated [32, 33]. Currently, it is agreed that ckit^+^CSCs release paracrine factors that, rather than the cells themselves, stimulate endogenous regenerative pathways, thus promoting myocardial regeneration and improved cardiac function [34, 35]. Nevertheless, the molecular mechanism activated in these cells and responsible for their paracrine effect on cardiomyocytes following transplantation in the infarcted heart is not well known.

Following myocardial injury, necrotic cardiomyocytes release DAMPs that, in leukocytes, trigger innate immune signaling pathways [4] and activate NFkB, leading to the IRR, that is involved in the protection and repair of the injured tissue. Therefore, leukocytes play a critical role in regulating the inflammatory and reparative response that follows MI. In our *in vitro* model, we found that cardiac ckit^+^CSCs can activate a gene response similar to that present in activated leukocytes. Accordingly, hypoxic conditions induced in our cells the activation of HIF-1 α, allowing them to adapt to the reduced oxygen level through the transcription of angiogenic factors and the switch from oxidative to glycolytic metabolism [10, 36]. Indeed, we detected an increased mRNA expression of VEGF and EPO following 6, 24 and 48 hours of hypoxia and a higher expression of GLUT1 and HK2. Interestingly, in a recent study, it has been demonstrated that, *in vitro*, treatment of bone marrow-derived angiogenic cells (BMDACs) with an inducer of HIF resulted in a metabolic reprogramming of these cells as reflected by the increased expression levels of glucose transporters, metabolic enzymes and pH regulators [37]. These alterations in metabolism allowed the survival of pre-treated BMDACs in the ischemic hindlimb and strengthen the concept that metabolic and cell survival pathways are closely related [38]. For instance, it is increasingly recognized that HK2 functions also as a protective signaling molecule. Specifically, in the heart, cardiac-specific HK2 overexpression prevents maladaptive hypertrophy by decreasing ROS accumulation [39]. In our experimental setting, we also found that hypoxia affects the expression of DAMP’s receptors. In particular, we observed that TLR2 and TLR4 were activated in ckit^+^CSCs after 24 and 48h of treatment and RAGE showed a significant mRNA up-regulation mainly after 6h of hypoxia and increased expression of protein levels at 24 and 48h.

The receptors for DAMPs, firstly localized in tissue resident leukocytes [40, 41], and later described in many other cells and tissues, under stress conditions, can be induced and expressed *de novo* in resident or recruited stem cells and pluripotent undifferentiated progenitors from tissues [42, 43]. For instance, mesenchymal stem cells (MSCs) express TLRs [44, 45] and upon their stimulation, paracrine factors are released and may boost or reduce their immunosuppressive capacities [46]. In the setting of I/R injury, two different studies suggested that TLR2 presence and activation on injected MSCs could be essential for myocardial recovery [47] while TLR4 activation would have deleterious effects [48]. Nevertheless, it should be noted that since both studies used an I/R injury in an isolated rat heart model, no immune system was present and therefore the effect of TLR2 and TLR4 activation on immunomodulation by MSC remains unclear.

Interestingly, in a prior study, we have already demonstrated that cardiac ckit^+^ cells express RAGE and their regenerative responses, elicited by intramyocardial injection of exogenous High Mobility Group Box-1 protein (HMGB1) following acute MI, are mediated, at least in part, by the interaction HMGB1-RAGE [49].

Our findings have shown a significant up-regulation of P2X7R mRNA in ckit^+^CSCs 48h following hypoxia. The purinergic P2X7R is involved in the migration and differentiation of different stem cells [50-52] and represents an essential pro-survival signal in embryonic stem cells [53]. In a model *ex vivo* of rat heart, agonists of this receptor are responsible for the release of cardioprotectants by ischemic pre- and post-conditioning [54].

Moreover, we observed that the mRNA expression levels of the inducible enzymes COX2 and NOS2 were up-regulated by hypoxia at all three time points in ckit^+^CSCs and that COX-2 protein expression was increased following 24h of hypoxia. Interestingly, Dr. Bolli’s group, using a molecular genetic approach and a model of MI, has demonstrated that the cardioprotection afforded by NOS2 gene therapy is mediated by COX2 up-regulation via NFkB activation [55]. In particular, up-regulation of COX2 determines an increase in myocardial production of two cytoprotective prostanoids [56].

The long pentraxin PTX3 is expressed in the heart under inflammatory conditions and has a cardioprotective role in acute myocardial infarction in mice [57]. More recently, it has been demonstrated that PTX3 deficiency is associated with increased atherosclerosis, macrophage accumulation and inflammation in the atherosclerotic lesions suggesting a modulation by PTX3 of the vascular associated inflammatory response [58]. Together these observations support the possibility that PTX3 may exert a cardiovascular protective effect through the modulation of the immunoinflammatory balance. Our results showed that PTX3 expression is influenced by hypoxia only at the earliest time point following hypoxia.

Finally, we detected a significant up-regulation of the receptor for CXC chemokines CXCR4 in ckit^+^CSCs after 48h of hypoxia. Hypoxic preconditioning of ckit^+^ cardiac progenitor cells improves their survival following MI [59] by inducing CXCR4 expression. Further, CXCR4 overexpression in MSCs attenuates cardiac remodeling after MI and this cardioprotective effect is mediated by the release of the antifibrotic enzyme MMP9 [60]. It is noteworthy that in our *in vitro* experiments, we detected a significant increase in the mRNA expression of MMP9 and a significant decrease in the mRNA expression of the MMP inhibitor TIMP4 in hypoxic ckit^+^CSCs compared to normoxic cells.

The activation of NFkB mounts a regenerative response by inducing genes that encode growth factors with the potential to mediate different cardioprotective effects. Our cells secreted high levels of IGF-1 and HGF under hypoxic conditions. These secreted paracrine factors have been demonstrated to exert beneficial effects on cell survival and cardiac remodeling. For instance, myocardial injury induced by cardiotoxin injection in transgenic mice with cardiac-restricted mIGF-1 expression determined restoration of cardiac function [61]. This effect was mediated, in part, by modulation of the inflammatory response. Specifically, the mRNA of IL10, a potent anti-inflammatory cytokine, was found to rapidly increase in transgenic hearts 24 hours after cardiac injury and this increase was even higher at 1 week [61]. It should be noted that IL10 has been showed to attenuate left ventricular remodelling after MI by reducing inflammation-mediated fibrosis [62]. Most importantly, transplantation of bone marrow mononuclear cells in infarcted mouse hearts led to the secretion of significant amounts of IL10 that were associated to decreased reactive hypertrophy and myocardial collagen deposition. These effects resulted in a significant improvement in cardiac function [63]. Interestingly, in our *in vivo* model, we detected higher levels of circulating IL10 in mice at 3 days following ckit^+^CSC transplantation compared to controls and, at the same time point, this result was confirmed by IL10 enhanced protein expression in the infarcted treated hearts. Further, in these mice, we also detected an increase in the phosphorylation of STAT3 whose signaling has been involved in IL10-mediated improvement in heart function in the MI model [62]. In the same mice, at 3 days but also at later time points, we also found decreased levels of circulating TGF β1 and, at 1 week, a reduced expression of collagen I in the border zone of treated infarcted hearts. The attenuation in LV remodeling in transplanted infarcted hearts compared to controls was also supported by up-regulation of CSRP3 and down-modulation of the β-MHC/α-MHC ratio, both indicative of a decrease in hypertrophy [64-66]. All these results were confirmed by an attenuated LV chamber dilation and wall stress in the heart of ckit^+^CSC transplanted mice compared to controls.

IL10 negatively affects cardiac remodelling also by attenuating apoptosis [67]. These data are in accordance with our results that showed the induction of autophagy and the reduction of apoptotic rate in infarcted hearts 3 and 7 days after ckit^+^CSC transplantation, respectively. Importantly, an antiapoptotic effect on cardiomyocytes is also exerted by HGF [68] and transplantation of MSCs overexpressing HGF in a mouse model of MI was associated with less cardiomyocyte apoptosis [69].

As previously discussed, growing evidence suggests that stem cell therapy following MI could improve cardiac function not by differentiation into cardiomyocytes and vascular cells but most likely by the production of paracrine factors which have beneficial effects [35] and could influence the immune system. In particular, it has been demonstrated by several reports that stem cell therapy could activate a subset of reparative macrophages [70]. For instance, transplantation of MSCs into the infarcted heart contribute to the recovery of cardiac function, in part, by switching macrophages from a pro-inflammatory to an anti-inflammatory and reparative phenotype through secreted factors such as IGF-1 and IL10 [46, 71-73]. Thus, a possible scenario is that ckit^+^CSCs, following transplantation in a hypoxic environment as the infarcted heart, exert cardioprotective effects by modulating the activation of macrophages via a paracrine mechanism. Indeed, in a very recent paper, it has been postulated that cardiac ckit^+^ progenitors originate from a cardiac myeloid lineage, i.e. macrophage progenitor cells [74] suggesting the potential of ckit^+^ progenitors to display functional characteristics of macrophages. Importantly, the beneficial effects of ckit^+^CSC treatment were confirmed by enhanced cardiac function.

In conclusion, in this study we have showed that cardiac ckit^+^CSCs adapt to a hypoxic environment through the activation of pathways similar to that one present in leukocytes and activate a reparative response that leads to the release of different paracrine factors.

## MATERIALS AND METHODS

### Animal model and *in vivo* study

Myocardial infarction (MI) was induced by coronary artery ligation in BalbC female mice 8 weeks old (20 gr body weight), as previously described [49]. Briefly, thoracotomy via the third left intercostal space was performed in mice under anesthesia (100 mg/kg ketamine and 1mg/kg acepromazine) and mechanically ventilated. Then the left coronary artery was ligated and 5X10^4^ ckit^+^CSCs cells in 10 μL PBS solution were injected through a 32-gauge needle. Four injections (2.5 μL per injection) were performed in the ventricular wall bordering the viable myocardium. Control infarcted mice were injected with 10 μL of PBS. Sham operated mice were treated similarly, except that the ligature around the coronary artery was not tied. Animals were sacrificed 3, 7, 14 and 21 days after surgery.

Hearts were processed in 4 different ways: 1) hearts were excised and the border and infarcted regions of infarcted mice were collected and stored at –80°C to be processed for protein extraction; specifically, the infarcted area was recognized as a pale zone caused by gross necrosis of the myocardium, due to interruption of the blood supply to the area while the border zone was identified as the area between the end of necrosis and the septum; 2) the abdominal aorta was cannulated and the heart was arrested in diastole with CdCl_2_, perfused retrogradely with 10% (vol/vol) formalin, embedded in paraffin and sectioned (3 μm thickness); 3) hearts were excised, perfused with Tyrode solution to remove blood and frozen in liquid nitrogen. Cryosections (5 μm thickness) were air-dried for 20 min; 4) the hearts of mice that did not undergo any surgery were used to isolate cardiac-derived cells by collagenase digestion of the left ventricle (LV).

All experimental procedures complied with the Guidelines of the Italian National Institutes of Health, with the Guide for the Care and Use of Laboratory Animals (Institute of Laboratory Animal Resources, National Academy of Sciences, Bethesda, MD) and were approved by the Institutional Animal Care and Use Committee.

### Evaluation of myocardial function

For hemodynamic studies, mice were anesthetized with chloral hydrate (400 mg/kg body weight) and the right carotid artery cannulated with a microtip pressure transducer (Millar 1.4F) [49].

### Cell isolation and culture

Cardiac-derived cells were isolated from BalbC female mice at 2–3 months of age by collagenase digestion (280 U/mL; Worthington, Lakewood, N.J., USA) through the coronary arteries and myocyte were removed by centrifugation. Cells were cultured in F12:DMEM 1:1 supplemented with 10% fetal bovine serum and expanded. Ckit^+^ cells were selected with immunomagnetic CD117 microbeads, mouse (Miltenyi Biotec) (Supplementary Figure 2) and were expanded in Ham’s F12 medium supplemented with 10% fetal bovine serum (FBS), penicillin-streptomycin and Erythropoietin (Sigma-Aldrich) 2,5 mg in 500 ml [75].

### Hypoxia

Hypoxic conditions were achieved by incubating ckit^+^CSCs in a humidified incubator Galaxy 14S (Eppendorf) at 37 °C with 5% CO_2_ and 1% O_2_ for 6, 24 and 48 h.

### FACS analysis

For FACS analysis, ckit^+^CSCs were fixed in 4% paraformaldehyde for 15 minutes at room temperature and incubated with anti-CD45 antibody (BD Pharmingen) or anti-ckit antibody (Santa Cruz). Isotype control was performed (mouse polyclonal IgG; Santa Cruz Biotechnology). Specifically, cells were incubated with the primary antibody for 45 minutes at 37°C. Flow cytometry was performed with FACSAria (Becton Dickinson, San Jose, CA) instrument. Cellular debris and aggregates were gated out based on forward and side scatter. Gating on the signal of the nuclear stain DAPI was employed to exclude additional artifacts. Isotype-matched negative controls were utilized to define the threshold for each specific signal and establish the appropriate gate for positive cells. Data were analyzed with Kaluza Flow Cytometry Analysis software [76].

### mRNA isolation and real time RT-PCR

Total RNA was extracted from ckit^+^CSCs following 6, 24 and 48h of normoxia and hypoxia (n=3/group). Quality of RNA was checked using the Agilent 2100 Bioanalyzer and nanodrop 1000. Then mRNA was quantified with ABI Prism 7000 SDS (Applied Biosystems). Real-time RT-PCR amplification was carried out in the 7900 Real-time PCR system (Applied Biosystems, CA, USA) using Real Time SyBr Green qPCR Superscript (Invitrogen, Milan, Italy). All reactions were carried out in triplicate. Relative expression was calculated using the comparative Ct method (2–[delta] [delta] Ct) [77]. The mean was calculated, and possible significant differences were analyzed using Student’s *t* test. *P* < 0.05 was considered significant.

The primers used are reported in Supplementary Table 1.

### Western blot analysis

Proteins from cardiac tissue or ckit^+^CSCs were homogenized and extracted with RIPA buffer (10 mM Tris-HCl pH 7.4, 150 mM NaCl, 1% NP40, 1% Deoxycolic acid, 0.1% SDS and 10% Glycerol) containing proteases and phosphatases inhibitors (2 mM phenylmethylsulfonyl fluoride, 100 U/ml of aprotinin, 10 μg/ml of leupeptin and pepstatin, 10 mM sodium fluoride, 20 mM sodium vanadate).

To separate cytoplasmic and nuclear fractions, cells were isolated using the nuclear exctraction kit from Active Motif (Carlsbad, CA), following manufacturer’s instructions. Briefly, cell were scraped off the plate in a phosphate–buffered saline/phosphatase inhibitor buffer using a rubber policeman. Cells were centrifuged 5 min at 500 rpm at 4°C and the supernatant discarded. The cell pellet was lysed in hypotonic buffer for 15 min in ice followed by centrifugation (30 s at 14 000 rpm at 4°C). The supernatant (cytosolic fraction) was transferred to a new tube, whereas the pellet (nuclear fraction) was lysed in a lysis buffer in the presence of protease inhibitors. Protein concentration of the fractions was determined by the Bradford assay (Bio–Rad, Hercules, CA).

Equal amounts of total cellular proteins (100 μg/lane) were resolved by SDS-polyacrylamide gel electrophoresis and transferred to nitrocellulose membrane (Amersham Pharmacia Biotech, Little Chalfont, UK). Membranes were probed with rabbit monoclonal anti HIF-1 α (Cell Signaling Technology, Danvers, MA), goat polyclonal anti HXK2 (Santa Cruz Biotechnology, Dallas, TX) rabbit polyclonal anti CAIX (Thermo Fisher Scientific, Whaltam, MA), rabbit polyclonal anti NFkB p65 (Santa Cruz Biotechnology), rabbit monoclonal Phospho–NFkB p65 (Cell Signaling Technology, rabbit monoclonal anti COX2 (Cell Signaling Technology), goat polyclonal anti RAGE (Santa Cruz Biotechnology), goat polyclonal anti CXCR4 (Santa Cruz Biotechnology), goat polyclonal anti alfa-MHC and mouse monoclonal anti beta-MHC (Santa Cruz Biotechnology), mouse monoclonal anti CSRP3 (Santa Cruz Biotechnology), rabbit polyclonal anti collagen I (Abcam, Cambridge, UK), rabbit polyclonal anti LC3B (Novus Biologicals, Littleton, CO), rabbit polyclonal anti Cdk4 (Santa Cruz Biotechnology), rabbit monoclonal anti Atg7 (Cell Signaling Technology), rabbit polyclonal anti Bax (Santa Cruz Biotechnology), rabbit polyclonal anti Cleaved Caspase-3 (Cell Signaling Technology) mouse monoclonal anti Bcl-2 (BD Biosciences Pharmingen, Sparks, MD), mouse monoclonal anti IL-10 (Santa Cruz Biotechnology), rabbit monoclonal anti Stat3 (Cell Signaling Technology) and rabbit polyclonal anti phosphor-Stat3 (Tyr705) (Cell Signaling Technology), followed by horseradish peroxidase-coupled secondary antibodies and developed by a chemiluminescence-based detection system (ECL, Amersham Pharmacia Biotech.).

### Blood sample collection and Luminex assay

Whole blood was collected just prior to euthanasia 3, 7 and 21 days after surgery in a 2ml polypropylene tube. After collection, the blood was allowed to clot by leaving it undisturbed at room temperature. The clot was removed by centrifuging at 2500 rpm (600 x g) for 15 minutes in a refrigerated centrifuge. The resulting supernatant was designated as serum. Following centrifugation, serum was immediately transferred into a clean tube. The sample was maintained at 2–8°C while handling. The previous step was repeated 2-3 times, until the serum appeared clear (without any trace of hemolyzed red blood cells). Serum was aliquoted and stored at -80°C for Luminex assay.

Cytokines were measured by Luminex technology using a customized panel by the Bio-Plex Pro Mouse Cytokine 23-plex, Group I (to analyze the following: IL-2, IL-4, IL-5, IL-10, IL-12p40, IL-17A, GM-CSF, INF- γ, TNF-α). The analysis was performed using 50 μl of the sample as manufacturer’s instruction. After the incubation with antibodies-activated magnetic beads, samples were washed using a Bio-Plex Pro TM Station (Bio-Rad) as described [78]. TGF β1 protein was analyzed using a Bio-Plex Pro TGF β assay (BioRad) according to manufacturer’s instructions with few modifications. Briefly, 12.5 μl of sample were first treated with 2.5 μl of 1N HCl, then neutralized using 2.5 μl of 1.2 N NaOH/0.5M HEPES. Samples were analyzed using a 1:16 final dilution. Then, they were incubated with antibodies-activated magnetic beads and washed using a Bio-Plex Pro TM Station (Bio-Rad). The quantification was carried out by using a Bio-Plex Array Analyzer (Bio-Rad) and with Bio-Plex Manager Software version 6.1. Results were expressed as pg/ml.

### Cardiac anatomy

After hemodynamic measurements, the abdominal aorta was cannulated, the heart was arrested in diastole and the left ventricular (LV) chamber was fixed at a pressure equal to the *in vivo* measured end-diastolic pressure. The LV intracavitary axis was measured, and three transverse slices from the base, mid-region and apex were embebbed in paraffin. The mid-section was used to measure LV thickness, chamber diameter and volume as described [49]. The chamber volume was calculated using the minimal and maximal luminal diameters at midregion with the longitudinal axis. Diastolic wall stress was determined from the wall thickness, chamber radius and left ventricular end diastolic pressure (LVEDP). Heart weight/Body weight ratio was also calculated. Tissue specimens were embedded in paraffin and sections were obtained for immunohistochemical studies.

### Hematoxylin-eosin (H&E) and Azan–Mallory staining

For hematoxylin-eosin (H&E) staining, murine sections were deparaffinized, rehydrated, placed first in haematoxylin and then in eosin for 5 minutes each, dehydrated and finally mounted. Several transverse sections were stained with Azan–Mallory to detect myocardial fibrosis.

### TUNEL assay

Terminal deoxynucleotidyltransferase (TdT)-mediated dUTP nick end labeling (TUNEL) assay was used for the detection of apoptosis [79] with ApoAlert DNA fragmentation kit (Clontech), following the protocol for formalin-fixed, paraffin embedded tissue sections mounted on glass slides. Briefly, 3 μm thickness sections were dewaxed in xylene, hydrated and rinsed in PBS. After paraffin removal, the slides were immersed in 0.85% NaCl and incubated at room temperature for 5 min, fixed in 4% formaldehyde/PBS (room temperature for 15 min) and washed twice in PBS. A rapid incubation in a 20 μg/ml Proteinase K solution (3-5 min) was performed and then sections were fixed again in 4% formaldehyde/PBS (this time, room temperature for 5 min). After that, sections were equilibrated using an equilibration buffer (room temperature for 10 min) and incubated in TdT incubation buffer (containing Nucleotide mix and TdT enzyme) for 1 h at 37°C in the dark. The tailing reaction was terminated by 2X SSC (room temperature for 15 min).

Nuclei were identified using SYTO 82 orange fluorescent nucleic acid staining (Molecular Probes, USA). Coverslips were mounted on anti-fade mountant (ProLong Diamond Antifade Mountant, Life Technologies) and sections were analyzed with a Zeiss microscope equipped for fluorescence.

Apoptotic rate defined the number of apoptotic cardiomyocytes on all cardiomyocytes per field. In order to avoid potential confounders deriving from the selection of fields with abnormal cardiomyocyte density, fields for the apoptotic rate count were selected in the peri-infarct area, defined as the zone bordering the infarct only where viable myocardium was prevalent and reparative fibrosis only marginal. Apoptotic rate in the peri-infarct region was calculated on 10 random fields, which virtually cover the entire peri-infarct area [80].

### Immunofluorescence analysis

For immunofluorescence, frozen tissue sections from the left ventricle were stained with a rabbit polyclonal anti LC3B (Novus Biologicals, Littleton, CO). Nuclei were identified using SYTO 82 orange fluorescent nucleic acid staining (Molecular Probes, USA). The cover-glasses were mounted and sections were analyzed with a LSM 510 confocal microscopy (Zeiss).

### ImmunoSorbent assay-ELISA

ELISA analysis for mouse IGF-1 and HGF (R&D Systems, Minneapolis, USA) were performed following the manufacturer’s instructions.

### Data collections and statistics

Results are presented as means ± error standard or mean±SD when specified. Statistical significance between two measurements was evaluated by unpaired Student’s *t* test and multiple comparisons was performed using 1-way analysis of variance and the Tukey test. A probability value of p<0.05 was considered significant. Data were analyzed using Prism software (version 7.0, GraphPad, San Diego, California).

## SUPPLEMENTARY MATERIALS FIGURES AND TABLE




