# Supplementary material for: Molecular mechanisms of cardioprotective effects mediated by transplanted cardiac ckit+ cells through the activation of an inflammatory hypoxia-dependent reparative response
Source: Oncotarget. 2017 Dec 6;9(1):937–57. doi: 10.18632/oncotarget.22946 (PMC5787525; doi:10.18632/oncotarget.22946)
Supplement: Supplementary file 1 [file oncotarget-09-937-s001.pdf]

# Molecular mechanisms of cardioprotective effects mediated by transplanted cardiac ckit+ cells through the activation of an inflammatory hypoxia-dependent reparative response

## SUPPLEMENTARY MATERIALS

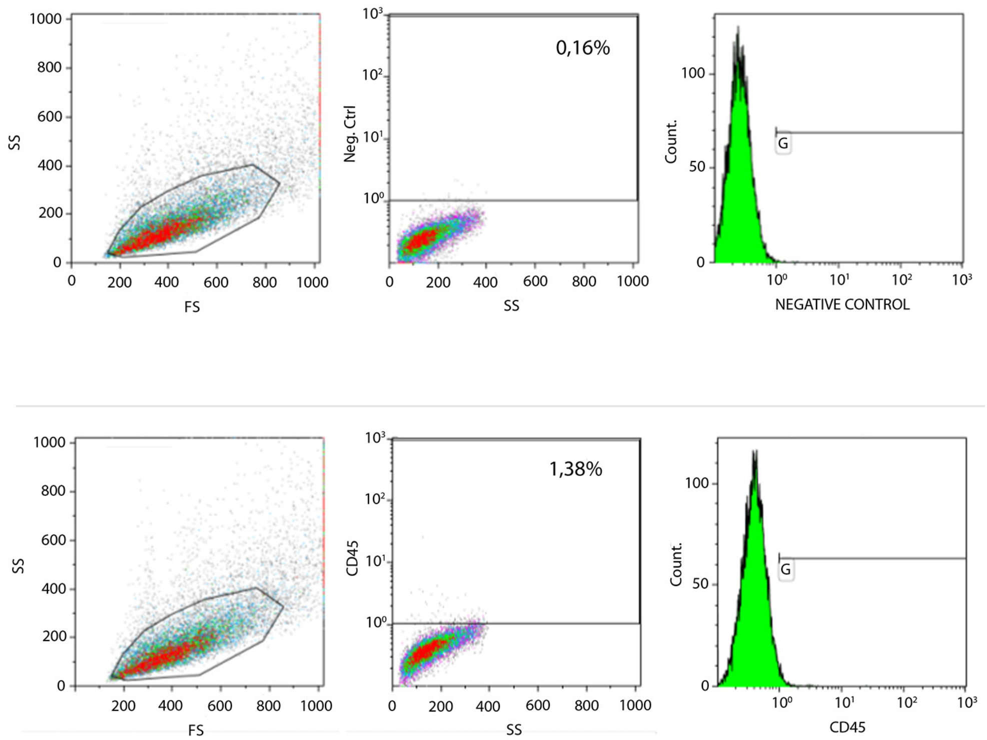


**Supplementary Figure 1: ckit$^+$CSCs do not express the hematopoietic marker CD45.** Scatterplots of ckit$^+$CSCs; the stem cell antigen ckit is expressed together with CD45. Negative isotype control is also shown.

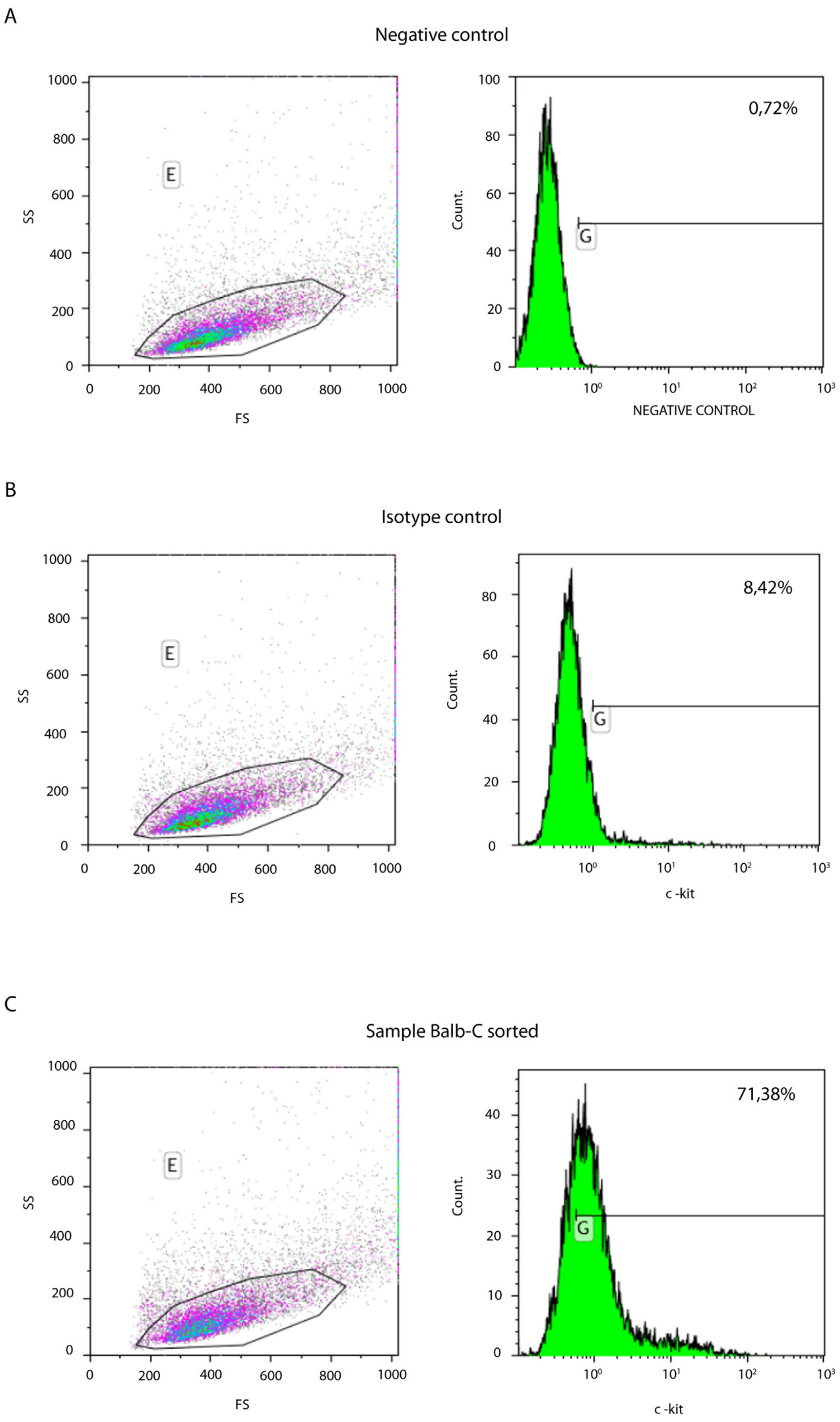


**Supplementary Figure 2: Characterization and growth properties of CSCs.** Distribution of c-kit expression in Isotype **(A)** and Negative control **(B)** preparation. c-kit expression **(C)** in sorted balb-c cells.

**Supplementary Table 1: Primer sequences for real time RT-PCR.**

**See Supplementary File 1**
