# Supplementary material for: Molecular mechanisms of cardioprotective effects mediated by transplanted cardiac ckit+ cells through the activation of an inflammatory hypoxia-dependent reparative response
Source: Oncotarget. 2017 Dec 6;9(1):937–57. doi: 10.18632/oncotarget.22946 (PMC5787525; doi:10.18632/oncotarget.22946)
Supplement: Supplementary file 2 [file oncotarget-09-937-s002.docx]

| HIF1A_mm_fw | gcactagacaaagttcacctgaga |
| --- | --- |
| HIF1A_mm_rev | cgctatccacatcaaagcaa |
| VEGFa_mm_fw | caggctgctgtaacgatgaa |
| VEGFa_mm_rev | gctttggtgaggtttgatcc |
| EPO_mm_fw | tctgcgacagtcgagttctg |
| EPO_mm_rev | cttctgcacaacccatcgt |
| Glut1_mm_fw | gaccctgcacctcattgg |
| Glut1_mm_rev | gatgctcagataggacatccaag |
| NFkB_mm_fw | ctggcagtccttctcaaagc |
| NFkB_mm_rev | tccaggtcatagagaggctca |
| Rage_mm_fw | agtcagaggaagcggagatg |
| Rage_mm_rev | aaggaggaattgggatggaatg |
| Cox2_mm_fw | caagcagtggcaaggcctcca |
| Cox2_mm_rev | ggcacttgcattgatggtggct |
| Nos2_mm_fw | ctttgccacggacgagac |
| Nos2_mm_rev | cattgtactctgagggctgac |
| Tlr2_mm_fw | accgaaacctcagacaaagc |
| Tlr2_mm_rev | agcgtttgctgaagaggact |
| Tlr4_mm_fw | ggactctgatcatggcactg |
| Tlr4_mm_rev | ctgatccatgcattggtaggt |
| P2rx7_mm_fw | aagtgcagacgctgtgtcc |
| P2rx7_mm_rev | gggaagaaaattgctgtttcac |
| Ptx3_mm_fw | cgctgtgctggaggaact |
| Ptx3_mm_rev | gggaagaaaattgctgtttcac |
| Cxcr4_mm_fw | tggaaccgatcagtgtgagt |
| Cxcr4_mm_rev | gggcaggaagatcctattga |
| MMP2_mm_fw | taacctggatgccgtcgt |
| MMP2_mm_rev | ttcaggtaataagcacccttgaa |
| MMP9_mm_fw | acgacatagacggcatcca |
| MMP9_mm_rev | gctgtggttcagttgtggtg |
| Timp3_mm_fw | cacggaagcctctgaaagtc |
| Timp3_mm_rev | tcccacctctccacaaagtt |
| Timp4_mm_fw | agggagagcctgaatcatca |
| Timp4_mm_rev | gcactgcatagcaagtggtg |
| GUSB_mm_fw | ctctggtggccttacctgat |
| GUSB_mm_rev | cagttgttgtcaccttcacctc |
